# Montelukast deprescribing in outpatient specialty clinics: A single center cross-sectional study

**DOI:** 10.1016/j.rcsop.2024.100509

**Published:** 2024-09-17

**Authors:** David C. Foote, Jamie L. Miller, Grant H. Skrepnek, Stephen Neely, Kiya Bennett, Paul M. Boylan

**Affiliations:** a Department of Experiential Education, The University of Oklahoma Health Sciences College of Pharmacy, 1110 N Stonewall Ave, CPB 139, Oklahoma City, OK 73117, United States of America; b Department of Pharmacy: Clinical and Administrative Sciences, The University of Oklahoma Health Sciences College of Pharmacy, 1110 N Stonewall Ave, O'Donoghue Research Building #4417, Oklahoma City, OK 73117, United States of America; c Department of Pharmacy: Clinical and Administrative Sciences, The University of Oklahoma Health Sciences College of Pharmacy, 1110 N Stonewall Ave, CPB 207, Oklahoma City, OK 73117, United States of America; d Office of Instruction, Assessment, and Faculty and Staff Development, The University of Oklahoma Health Sciences College of Pharmacy, 1110 N Stonewall Ave, CPB 222, Oklahoma City, OK 73117, United States of America; e Department of Pharmacy: Clinical and Administrative Sciences, The University of Oklahoma Health Sciences College of Pharmacy, 1110 N Stonewall Ave, CPB 219, Oklahoma City, OK 73117, United States of America; f Department of Pharmacy: Clinical and Administrative Sciences, The University of Oklahoma Health Sciences College of Pharmacy, 1110 N Stonewall Ave, CPB 239, Oklahoma City, OK 73117, United States of America

**Keywords:** Montelukast, Deprescribing, Drug labeling, Hospital outpatient clinics, Black box warnings

## Abstract

**Objective:**

To identify and evaluate montelukast deprescribing in outpatient specialty clinics.

**Methods:**

This was a single-center, retrospective, cross-sectional study conducted at an academic health system in the southern US including 21 specialty clinics. Subjects included adults ≥18 years with an active prescription for montelukast who attended at least one appointment in pulmonology, otolaryngology, or neurology outpatient specialty clinics between January 1, 2021 to December 31, 2022. Patients <18 years and those with diagnoses of uncontrolled asthma or allergic rhinitis were excluded. Outcomes assessed included the frequency and period prevalence of montelukast deprescribing, defined by a documented montelukast discontinuation within the medical record, and evaluation of reasoning for discontinuation mentioned in visit notes.

**Results:**

There were 1152 patients who met inclusion criteria. Of these, 43 (3.7 %) experienced a montelukast deprescribing event: 18 (41.9 %) in neurology, 13 (30.2 %) in otolaryngology, and 12 (27.9 %) in pulmonology. Documented reasons for deprescribing were only available for 11 patients (25.6 %); reasons for deprescribing included patient-provider shared decision-making regarding the Black Box Warning [*n* = 5 (11.6 %)], inadequate treatment response [*n* = 3 (7.0 %)], suicidal thought development [*n* = 1 (2.3 %)], adverse drug event [*n* = 1 (2.3 %)], and pregnancy planning [*n* = 1 (2.3 %)].

**Conclusion:**

Montelukast deprescribing rates were less than 5 % in outpatient specialty clinics. Factors associated with montelukast deprescribing beget further investigation.

## 1 Introduction

The leukotriene receptor antagonist, montelukast, was the 17th most-prescribed medication by-volume in the United States (US) in 2022.^1^ Though montelukast possesses US Food and Drug Administration (FDA) approval to treat asthma and allergic rhinitis in pediatric and adult populations,^2^ it is listed as alternative therapy for these indications in current clinical practice guidelines due to lower effectiveness than comparable medications.3., 4, 5 In 2020, the FDA added a Black Box Warning to montelukast's label concerning potential increased risk for neuropsychiatric symptoms including suicidal intent and actions.^6^ The FDA Expert Panel explained their reasons for adding the warning were to increase prescriber awareness, promote medication monitoring, and address stakeholder concerns.^7^ Patient and caregiver testimonies were among the documented factors behind the FDA's decision, specifically patients' comments that prescribers did not engage in shared decision-making with them regarding montelukast's benefit-risk profile. The pharmacologic mechanism by which montelukast may increase the risk for neuropsychiatric events is unknown, though may include leukotriene dysregulation in the central nervous system, the tryptophan/kynurenine pathway, or montelukast's quinoline structure.^7^^,^^8^

Given the recent addition of neuropsychiatric events as a Black Box Warning for montelukast, re-evaluation of opportunities for deprescribing is warranted. Based upon the Agency for Healthcare Research and Quality (AHRQ) Medical Expenditure Survey Panel (MEPS) from 2022, community pharmacies reported dispensing montelukast to 6.8 million patients in the US.^1^ Since 2013, this represents an absolute increase of almost 2 million additional patients per year (up from 5.1 million patients per year) who have been prescribed montelukast. This may indicate a large and increasing number of patients are exposed to montelukast each year and, thus, deprescribing opportunities may exist to prevent montelukast's neuropsychiatric adverse drug events.^1^^,^^9^ In the five weeks following the Black Box Warning announcement, public interest in montelukast (via Google Trends search volume) increased over 75 % compared to the previous 3 years.^10^ From 2020 to 2022, voluntary adverse event reporting to the US FDA Adverse Events Reporting System Public Dashboard for adolescents 11 to 17 years of age taking montelukast increased between 28.2 % and 233 % (compared to the 2018–2020 reporting period) across mental health symptom categories including anxiety, depression, attention deficit hyperactivity disorder, and suicidal behavior.^11^

The practice of deprescribing has been identified as a high priority for inclusion in AHRQ's Making Healthcare Safer Report of 2023.^12^ Specific interventions for deprescribing have been investigated, with recommended methods including medication reviews (some of which may be guided by specific criteria including Screening Tool of Older Persons' Prescriptions criteria).^13^ Methods for deprescribing as supported by literature include clinical decision support tools, “direct-to-patient” educational interventions, and the collection of a complete and accurate medication history.^13^, ^14^, ^15^ The literature surrounding deprescribing montelukast is limited and incongruous.^16^ Metzler et al.^17^ and Drummond et al.^18^ both evaluated treatment effectiveness with montelukast compared to other therapies, however, neither investigation assessed deprescribing in specialty clinics as these studies were conducted prior to the FDA's issuance of a Black Box Warning.

The incidence of montelukast-associated neuropsychiatric events may be as common as 1 event per 100 patients (i.e., sleep disturbances, depression, and agitation), as uncommon as 1 event per 1000 patients (i.e., memory or attention impairment), or rare as 1 event per 10,000 patients (i.e., hallucinations or suicidal behavior).^9^ However, given that approximately 7 million patients are prescribed montelukast each year, between 700 to 70,000 patients may experience a preventable adverse neuropsychiatric event.^1^^,^^9^ Evaluating the case characteristics and opportunities for providers to deprescribe montelukast may lead to avoidance of preventable adverse effects. The purpose of this study was to assess the frequency and period prevalence of montelukast deprescribing within outpatient specialty clinics in an academic health system in the southern US.

## 2 Methods

### 2.1 Study design and setting

This was a single-center, retrospective, cross-sectional study at a 500-bed academic medical center at The University of Oklahoma Health Sciences that included 21 specialty clinics. The cross-section period was January 1, 2021 to December 31, 2022. Patients attending an appointment at pulmonology, neurology, or otolaryngology specialty clinics during this timeframe provided the basis for the study's sample population. These specialty clinics were targeted given prescriber's accompanying knowledge of neuropsychiatric events and stake in the guidelines for both allergy and asthma, and previous data have shown specialists are 9 % more likely than generalist prescribers to discuss Black Box Warnings with patients.^19^ Primary care clinics (i.e., internal or family medicine) were excluded given previous data suggesting that 14 % of essential information, including contraindications mentioned in Black Box Warnings, is missed during primary care encounters.^20^^,^^21^ Data reporting adhered to the Strengthening the Reporting of Observational Studies in Epidemiology (STROBE) checklist^22^ and was approved by the affiliated institutional review board (protocol #16395, Expedited Category 5, October 9, 2023).

### 2.2 Population

Patients were eligible for inclusion if they were 18 years of age or older at their first qualifying specialty clinic visit and had an active prescription for montelukast on their home medication list for the anteceding 3 months. Patients were excluded if they were under 18 years of age, were diagnosed with unstable asthma (i.e., exacerbation or hospitalization within 12 months), or had allergic rhinitis symptoms that interfered with daily activities.^3^^,^^4^^,^^23^ Medical records were identified from the affiliated clinical research data warehouse and charts were reviewed for inclusion and exclusion by one investigator (DF) following retrospective chart review best practices.^24^

### 2.3 Outcomes

The outcome of interest was the frequency and period prevalence of deprescribing of montelukast within the specialty clinics. Deprescribing was defined as a removal of the prescription from the patient's medical record without any further documentation of being prescribed or dispensed in the future.^25^ A second outcome was to identify clinical documentation concerning reasons for montelukast deprescribing.

### 2.4 Data collection

Data were collected via the electronic medical record, Epic (Epic Systems, Verona, WI). Variables including patient demographics and detailed visit information were collected from the data identified by the affiliated data research warehouse. Montelukast deprescribing information, allergies, history of suicidal thoughts, and screening tools for patient mood were collected using manual chart review of patient care notes by one investigator (DF).

### 2.5 Statistical methods

Data importation and management were conducted via Microsoft Excel (Microsoft Corporation, Redmond, WA). Descriptive statistics included mean (standard deviation), median (interquartile range), counts, and frequencies (percentage). The period prevalence of deprescribing was reported overall and by the provider's department over the study period of two years (i.e., 2021 to 2022). Data were analyzed using SAS software, version 9.4 (SAS Institute Inc., Cary, NC).

## 3 Results

Between January 1, 2021 and December 31, 2022, there were 1500 records meeting initial eligibility criteria identified by the data warehouse; 186 were excluded due to patient age less than 18 years and 162 were excluded due to lacking a qualifying specialty clinic visit or montelukast was unreported on their home medication list. Thus, 1152 patients were included in the cross-sectional cohort. Table 1 presents the demographic characteristics. Summarily, the average patient age was approximately 56 ± 17.3 years and a majority were female (*n* = 801, 69.5 %), White race (*n* = 918, 79.6 %), and metropolitan-residing (*n* = 963, 83.6 %).

**Table 1:** Characteristics of Montelukast Prescribing within Specialty Clinics, 2021–2022.

**Characteristic**	**Overall *N*** **=** **1152**
Female Sex	801 (69.5 %)
Age, years	56.5 ± 17.3
Number of qualifying visits	5 ± 8

Residence	
Metropolitan	963 (83.6 %)
Micropolitan	109 (9.5 %)
Rural	34 (3 %)
Small town	34 (3 %)
Unreported	12 (1 %)

Race	
American Indian or Alaska Native	33 (2.9 %)
Asian	22 (1.9 %)
Black or African American	167 (14.5 %)
White	918 (79.7 %)
Unreported	7 (0.6 %)
Other	5 (0.4 %)

Ethnicity	
Not Hispanic or Latino	1106 (96 %)
Hispanic or Latino	38 (3.3 %)
Unreported	8 (0.7 %)

Data reported as frequency (percentage) or mean ± standard deviation.

Deprescribing events across the specialty clinics and the documented reasons for montelukast deprescribing appear in Table 2. Forty-three patients (3.7 %) were involved in a deprescribing event across the following specialty clinics: 18 (41.9 %) in neurology; 13 (30.2 %) in otolaryngology; and 12 (27.9 %) in pulmonology. Of the 43 deprescribing events, 32 (74.4 %) occurred following an electronic medication reconciliation during the appointment, but lacked narrative documentation for montelukast deprescribing. The reasons for the remaining deprescribing events included: patient-provider shared decision-making regarding the Black Box Warning [*n* = 5 (11.6 %)]; inadequate treatment response [*n* = 3 (7.0 %)]; development of suicidal ideation [*n* = 1 (2.3 %)]; adverse drug event [*n* = 1 (2.3 %)]; and pregnancy planning [*n* = 1 (2.3 %)].

**Table 2:** Montelukast Deprescribing Events Across Specialty Visits, 2021–2022.

Deprescribing Reason	Pulmonology	Otolaryngology	Neurology	Total
Reason Undocumented^a^	6	9	17	32
Black Box Warning Risk-Benefit Discussion	5	0	0	5
Poor Efficacy	0	2	1	3
Suicidal Thoughts	1	0	0	1
Pregnancy Intent	0	1	0	1
Adverse Drug Event^b^	0	1	0	1
**Total**	12 (27.9 %)	13 (30.2 %)	18 (41.9 %)	*n* = 43

Data reported as frequency (percentage).

a Documentation in the medical record not provided.

b One patient developed fatigue.

## 4 Discussion

This single-center, retrospective, cross-sectional study of 1152 patients observed a 3.7 % period prevalence for deprescribing montelukast between 2021 and 2022 in specialty clinics including neurology, otolaryngology, and pulmonology. Among deprescribing events, almost 75 % of events lacked documentation as to the reason why montelukast was deprescribed. Approximately 12 % of montelukast deprescribing events occurred following the FDA's Black Box Warning guidance for patients and prescribers to engage in shared clinical decision-making and weigh the risks versus benefits in lieu of neuropsychiatric event risks.

The current study's results are among few reports to characterize and calculate the prevalence of montelukast deprescribing, and findings suggest this occurrence is infrequent. The Expert Panel discussion for the FDA Black Box Warning for montelukast reported a concern for the “cavalier” prescribing of the medication, as evidenced by it being among the top twenty most prescribed medications by-volume in the US during that time.^1^^,^^7^ In 2019, montelukast was prescribed to 7,429,725 patients and decreased to 6,825,788 patients in 2022, indicating an 8.1 % decrease in montelukast prescriptions since publication of the Black Box Warning.^1^ Results from this cross-sectional study are in concert with national trends and may indicate that montelukast deprescribing is uncommon and that efforts in outpatient specialty clinics are needed to assess the appropriateness of montelukast prescribing.

In the Black Box Warning for montelukast and accompanying patient testimonials during panel hearings, the US FDA specifically recommended engagement in shared decision-making to alert patients of possible changes in behavior or new symptoms that could be associated with adverse effects.^6^^,^^7^ Despite this specific recommendation, within the current study, only 5 patients in pulmonology clinics engaged in a documented discussion of risk-benefit regarding montelukast, resulting in deprescribing. This may suggest that, even within specialty provider clinics with the most attention to montelukast-specific outcomes and whose professional organizations publish clinical practice guidelines advocating against montelukast, appropriate deprescribing might not be performed.^4^^,^^5^ This somewhat mirrors pulmonologists' prescribing patterns in the year 2005 when the FDA added a Black Box Warning to long-acting beta agonist prescribing information contraindicating this class as monotherapy for the treatment of asthma; pulmonologists were significantly more knowledgeable than primary care providers (99.0 % vs 90.8 %, *p* < .001) regarding the new Black Box Warning, though only 35 % changed their prescribing habits because of the Warning.^19^ Overall, recommended system-level efforts to consider appropriate deprescribing of montelukast include computerized decision support systems, implementation of protocols, public awareness, patient empowerment, and pharmacist-driven education efforts.^26^, ^27^, ^28^ The FDA acknowledged that the addition of the Black Box Warning was among the last tools at their disposal to inform prescribers and to facilitate deprescribing.^7^

More in-depth investigation of the association of montelukast with neuropsychiatric events would be of benefit to patients and prescribers alike. Future guideline recommendations could also include stronger emphasis on the risks of neuropsychiatric events that could be associated with montelukast to reduce the number of patients being prescribed the medication. The implementation of these systematic and patient-focused changes are aims to increase the rates of deprescribing where appropriate. Conversely, the FDA may also be poised to de-label the Black Box Warning altogether, given research conducted after their 2020 warning suggests there may be no association between montelukast and self-harm attempts or events and that suicidal ideation may occur in as few as 1 in 10,000 patients treated.^9^^,^^29^, ^30^, ^31^ In the primary care setting, comprehensive medication reconciliations may include assessments of risks and benefits for each medication. Evaluation of possible neuropsychiatric adverse effects, including those identified in the Black Box Warning, should be performed during each visit and for every medication to appraise pharmacotherapy safety and efficacy; this may be accomplished by administering either the Patient Health Questionnaire 2- or 9-item instruments, as recommended by the US Preventive Services Task Force.^32^ Annual wellness visits may present as an opportunity for this routine assessment, which may prompt the management of neuropsychiatric illnesses.^33^^,^^34^

Due to newly-published, conflicting information in the literature suggesting that montelukast neither significantly nor clinically increases self-harm thoughts and attempts,^29^, ^30^, ^31^ providers may be hesitant to deprescribe montelukast. However, as stated previously, the intent of the Black Box Warning is to increase provider and patient awareness and encourage shared decision-making.^7^ Therefore, providers should nonetheless document that a discussion occurred with the patient, and the agreed upon plan to deprescribe or to continue montelukast should be documented.^14^^,^^15^ As observed in the findings of this study, documentation for the reason behind discontinuation was unavailable for almost 75 % of encounters. In the same respect, it is plausible that discussions took place, and the decision was made to continue therapy, but the discussion was not documented in the medical record. If the decision is made to continue therapy, then providers should document that education was provided and include discussion of potential adverse effects. Deprescribing documentation should be placed in emails, letters, or handouts to patients, and in telephone or progress notes in electronic medical records.^15^ Furthermore, inpatient providers may be well-positioned to evaluate benefits and risks in hospitalized patients who reported taking montelukast prior-to-hospitalization. Special consideration could be given to patients admitted with new onset or exacerbation of an underlying neuropsychiatric illness.^34^

### 4.1 Limitations

Despite findings, the current retrospective, single-center investigation of 1152 patients warrants both experimental studies and multi-site real-world evidence to evaluate the association of montelukast with neuropsychiatric events. Compared to the 2012–2015 National Ambulatory Medical Care Survey of asthma-related outpatient visits, our cohort may overrepresent White (79.7 % vs 63.5 %) and female (69.5 % vs 53.8 %) populations living with asthma.^35^ Due to the retrospective nature, this study relied solely on documentation for clinical justification of discontinuation or possible choices for continuation, both of which may have occurred without documentation in some cases. The health system studied does not include allergy specialty outpatient services and, thus, allergist deprescribing of montelukast warrants additional research. Additionally, the lack of access to confidential notes in the electronic health record could have limited the viewable documentation of neuropsychiatric events, especially in outpatient psychiatric clinics. Although an endorsed definition of deprescribing was utilized,^25^ it does not completely differentiate medication nonadherence from intentional deprescribing. Caution should be exercised in extrapolating findings from this study to other settings and patient populations.

## 5 Conclusions

Of 1152 patients on montelukast seen in qualified outpatient specialty clinics, 3.7 % experienced a montelukast deprescribing event. A continued need may be present for shared decision-making surrounding the risks and benefits of montelukast, for more detailed evaluation of those who would most benefit from montelukast deprescribing, and for effective strategies to increase appropriate montelukast use.

## Funding details

This work was not supported by funding.

## Disclosure statement

Paul M. Boylan and Grant H. Skrepnek report unrelated and unrestricted research grant funding to The University of Oklahoma Health Sciences from Pfizer, Inc. Dr. Skrepnek also reports unrelated research grant funding to The University of Oklahoma Health Sciences from Otsuka Pharmaceutical, Inc. and Janssen Scientific Affairs, LLC. Other authors declare that they have no known competing financial interests or personal relationships that could have appeared to influence the work reported in this paper.

## Presentation

This project was presented as a poster at the American Society of Health-System Pharmacists Midyear Clinical Meeting in December 2023 and a platform presentation at the Oklahoma Society of Health-System Pharmacists in May 2024.

## CRediT authorship contribution statement

**David C. Foote:** Writing – review & editing, Writing – original draft, Investigation, Conceptualization. **Jamie L. Miller:** Writing – review & editing, Validation. **Grant H. Skrepnek:** Writing – review & editing, Validation, Methodology. **Stephen Neely:** Software, Methodology, Formal analysis, Data curation. **Kiya Bennett:** Writing – review & editing, Validation. **Paul M. Boylan:** Writing – review & editing, Writing – original draft, Supervision, Resources, Project administration, Methodology, Conceptualization.

## Declaration of competing interest

The authors declare the following financial interests/personal relationships which may be considered as potential competing interests:

Paul M Boylan reports a relationship with Pfizer Inc. that includes: funding grants. Paul M Boylan reports a relationship with Pharmacy Times Office of Continuing Professional Education LLC that includes: speaking and lecture fees. Grant H Skrepnek reports a relationship with Pfizer Inc. that includes: funding grants. Grant H Skrepnek reports a relationship with Otsuka America Pharmaceutical Inc. that includes: funding grants. Grant H Skrepnek reports a relationship with Janssen Scientific Affairs that includes: funding grants. If there are other authors, they declare that they have no known competing financial interests or personal relationships that could have appeared to influence the work reported in this paper.
